# Does language matter? A case study of epidemiological and public health journals, databases and professional education in French, German and Italian

**DOI:** 10.1186/1742-7622-5-16

**Published:** 2008-09-30

**Authors:** Iacopo Baussano, Patrick Brzoska, Ugo Fedeli, Claudia Larouche, Oliver Razum, Isaac C-H Fung

**Affiliations:** 1 Department of Infectious Disease Epidemiology, St. Mary's Campus, Imperial College London, UK; 2 CPO-Piemonte, Novara, Italy; 3 Department of Epidemiology & International Public Health, School of Public Health, University of Bielefeld, Germany; 4 SER-Epidemiological Department, Veneto Region, Castelfranco (TV), Italy; 5 Department of Community Health and Humanities, Faculty of Medicine, Memorial University of Newfoundland and Labrador, Canada

## Abstract

Epidemiology and public health are usually context-specific. Journals published in different languages and countries play a role both as sources of data and as channels through which evidence is incorporated into local public health practice. Databases in these languages facilitate access to relevant journals, and professional education in these languages facilitates the growth of native expertise in epidemiology and public health. However, as English has become the *lingua franca *of scientific communication in the era of globalisation, many journals published in non-English languages face the difficult dilemma of either switching to English and competing internationally, or sticking to the native tongue and having a restricted circulation among a local readership. This paper discusses the historical development of epidemiology and the current scene of epidemiological and public health journals, databases and professional education in three Western European languages: French, German and Italian, and examines the dynamics and struggles they have today.

## Introduction

Epidemiology and public health have followed multiple lines of development in Western Europe in the past several centuries [1]. From the quarantine of ships introduced by powerful Italian city-states to the improvement in water supply and the sewage system in Victorian England, many schools of thought from diverse social and national contexts contributed to the development of various public health practices and the science of epidemiology [2]. The expansion of colonial empires gave birth to the discipline of tropical medicine [3], while the Industrial Revolution, and its accompanying urbanisation and impoverishment of the urban proletariat, brought into being social medicine and its concern for environmental and occupational health [2]. Spatial and temporal differences of these developments across Western Europe due to different national contexts are observed. While epidemiology had made much progress since the 19^th ^century in countries such as Great Britain [4], France [5] and Germany [6], in countries such as Italy it only really took off as an independent discipline after the Second World War [7].

Since the end of the Second World War, English has become the international language of scientific communication [8]. Academic journals of non-English-speaking countries struggle for survival and are under increasing pressure to switch to English [9]. However, this pressure is troubling, due to the context-specific nature of epidemiology and public health, as well as its plurisecular development in Western Europe [1]. Therefore, it is important for the understanding of the development and future of epidemiology to examine the academic literature and online resources available in Western European languages other than English, as well as both the opportunities and difficulties these journals face in the age of globalisation.

In this article, French, German and Italian have been chosen as representatives of non-English Western European languages. (For Portuguese and Spanish scenarios, please refer to articles by Barreto and Barata [10], and Williams et al. [11] in this thematic series). Through comparison between the three scenarios, this paper highlights how factors outside science (e.g. economic, social, political etc.) influence the development of epidemiology and public health journals, databases and professional education across languages.

To cover the world-wide Francophone community, French journals and online resources of Canadian and African origin are also included. However, as a limitation it should be mentioned that the collection of epidemiological journals presented here is based on the authors' knowledge and experience in the field as well as relevance of the journals (only those journals that frequently publish articles in epidemiology and public health are selected), and therefore the lists of epidemiological and public health journals are not meant to be exhaustive and authoritative. Apart from journals and databases, professional education in epidemiology in the respective languages is also discussed. Professional education is important as it brings into existence a community of epidemiologists that makes local epidemiology possible and forms the readership of local journals.

## Scenario 1: French

### Background: France

It might be difficult to believe that France was the pioneer in public health and epidemiology publishing, considering the abundance of such literature published in English today. Nevertheless, it was. The first journal of public health in any language was founded by French physicians in 1829 and was published in French. The *Annales d'hygiène publique et de médecine légale *has been the leader in this field for a long time [5]. Fifty years later, with the creation of the first two national public health organisations in 1877, three new journals focusing on public health and hygiene were created, namely the *Journal d'hygiène*, the *Bulletin de la Société de Médecine Publique *and the *Revue d'hygiène et de police sanitaire *[5].

France is recognised as the country that created the first model of public health as a complete and coherent discipline in the 19^th ^century, albeit through more academic means rather than a discipline in and of itself [2,5]. Even though health has been proclaimed as a right for every citizen in a modern democracy after the French Revolution (1789–1799), preventive medicine continued to be applied through local initiatives in villages and cities instead of through a larger, recognised movement. Contrary to England, France considered disease prevention as an intellectual and academic science, and this fact delayed the construction of disease prevention as a (practical) discipline until the end of the 19^th ^century [2].

However, this trend gradually changed with Louis Pasteur's discoveries related to bacteriology and microbiology. As a chemist, Pasteur discovered that organisms such as bacteria were responsible of the change of taste in beer and wine as well as milk. He demonstrated that bacteria could be removed by heating the liquid (pasteurisation) of each beverage [12]. He also created the first vaccine against rabies, and on the whole his work served to improve epidemiology and public health. In 1888, the "Institut Pasteur" opened its doors in Paris [13].

### Background: Québec

In Canada, the emergence of public health began late in the 19^th ^century with the birth of the Confederation in 1867, after which the provinces were not under the control of England anymore [14]. The creation in 1882 of the first Provincial Board of Health in Ontario marked the beginning of a recognised public hygiene movement. The only French province in Canada, Québec, followed four years later with the "Conseil d'hygiène de la Province de Québec". After the establishment of the Confederation, England continued to have a strong influence on Canada's decisions – e.g., its 1875 public health laws have strongly influenced Canada's general public health policies [14].

It is well known that Canada is divided linguistically into two communities with two official languages: English and French. The first recorded specialised journal on public health, *Public Health Journal*, began in 1910 and was published in both languages [15]. Between 1880 and 1910, a group of Québécois physicians created the first sanitary education journal (*Journal d'hygiène populaire*), whose mission was to educate to the population of Québec on proper tools for hygiene [16].

In the first decade of the 20^th ^century, Québec and the nine other Canadian provinces developed parallel health care systems. The particular linguistic profile of Canada probably encouraged and influenced Québec to develop its own competences as well as to take its own actions in public health. In addition, constant tensions between French and English-speaking Canadians within medical and public health communities also enabled Québec to develop this emergent science independently [15]. The creation of the first French-speaking hygiene school (University of Montréal) in North America in 1946 is one of the best examples of this dynamism [16].

### Journals in epidemiology and public health in French: a portrayal of France, Québec and francophone Africa

In France, there exists a notably larger network of epidemiologists and public health practitioners; for example, the French Language Epidemiologists Association founded in 1976 [17] includes epidemiologists from everywhere in France. This larger network and the history of France in the development of preventive medicine might explain the greater number of publications from France than those from Canada, as it is noticeable in Table 1. France obviously has more publications than either Canada or Africa, but unlike Canada, journals published in France are unilingual (except for the *Revue d'épidémiologie et de santé publique*). In addition, France also has an online public health database containing 28,047 accessible documents, as of December 5^th^, 2007 [18].

**Table 1:** Selection of French language journals relevant to epidemiology

**Title**	**English translation (for French title)**	**ISSN (Print/Electonic)**	**Epidemiological topics covered^1^**	**Official Impact factor 2006**	**Listed in Medline**	**Issues per year**	**First edition^2^**	**Country of publication**	**Official journal language**	**Notes of Language**	**URL**
***Category A: Journals covering (mainly non-clinical epidemiology)***											
*Archives de l'Institut Pasteur de Madagascar*	Archives of the Madagascar Pasteur Institute	0020-2495	Allergy- Vaccine-Immunology- Public Health	-	Yes	2	1954	Madagascar	French	French	
*Archives de l'Institut Pasteur de Tunis*	Archives of the Tunis Pasteur Institute	0020-2509	Public health epidemiology-Microbiology	-	Yes	4	1923	Tunisia	French	French	
*Bulletin de l'Académie nationale de médecine*	Bulletin of the National Academy of Medicine	0001-4079	Public Health Wide range of topics	0.323	Yes	9	1947	Netherlands*	French	French	
*Bulletin de l'Institut national de la santé et de la recherche médicale*	Bulletin of the National Health Institute and Medical Research	0553-2469	Public Health-Research	-	Yes	6	1964	France	French	French	
Cahier de santé publique	Public Health Book	1817-5538	Public Health Wide range of topics	-	No	†	-	Côte d'Ivoire	French	French	
*Concours medical*	Medical Contest	0010-5309	Public Health Wide range of topics	-	Yes	41	1879	France	French	French	
Dakar Médical	Medical Dakar	0049-1101	Medicine- Tropical Medicine	-	Yes	2	1979	Senegal	French	Articles in French with summaries in French and English	
Le journal africain de la médecine et des sciences médicales	African journal of medicine and medical sciences	0309-3913	Public Health-Medicine Wide range of topics	-	Yes	4	1976	Nigeria	French/English	Articles in French or English with summaries in both languages	
Le Mali médical	Mali Medical	0464-7874/1993-0836	Medicine Wide range of topics	-	Yes	2	1963	Mali	French	French	
Médicine d'Afrique noire	Black Africa Medicine	0047-6404	Education- Medical-Graduate	-	Yes	11	1954	Senegal	French	French with summaries in English and French	
*Politiques de Santé (continuing Ruptures)*	Healthcare Policy	1715-6572	Public Health-Practice- Canadian-Wide range of topics	-	No	4	-	Canada	French/English	Articles in French and English, summaries and table of contents in English and French	
*Population et sociétés*	Population and Societies	0184-7783	Bulletin-Epidemiology- Social sciences- History of Medicine Wide range of topics	-	Yes	12	1968	France	French	French	
*Revue d'épidémiologie et de santé publique*	Epidemiology and Public Health	0398-7620	Journal – Education-Practice- Social Medicine- Health Research	0.592	No	6	1976	France	French/	Articles in French or English with summaries and table of content in both languages	
*Revue canadienne de santé publique*	Canadian Journal of Public Health	0008-4263	Public Health-Education- Practice-Canadian Public Health Wide range of topics	-	Yes	6	1943	Canada	French/English	Issues for 1943-59 have English title only. French title varies:*Revue canadienne d'hygiène publique*, 1960-69 Text chiefly in English, with some in French.	
*Revue Santé, Société et Solidarité*	Health, Society and Solidarity	1634-8176	Social sciences-Public Health- Wide range of topics	-	No	2	2002	Canada/France	French	French	
*Santé*	Health	1157-5999	Public Health-Environmental Health	-	Yes	4	1990	France	French	Articles in French; summaries and table of contents in English and French	
*Santé publique*	Public Health	0995-3914	Public Health Wide range of topics	-	Yes	6	1988	France	French	Articles in French; summaries in English and French. Formed by the union of Cahiers de l'Ecole nationale de la santé publique and Revue française de la santé publique	
***Category B: Medical journals occasionally covering (mainly clinical) epidemiology***											
*Développement et santé: revue de perfectionnement médical et sanitaire en pays tropical*	Development and Health: Journal of Medical learning and sanitary in tropical countries	0396-8014	Public Health – Medicine – Tropical Climate Wide range of topics	-	Yes	6	1976	France	French	French	
*Relevé des maladies transmissibles au Canada*	Canada communicable disease report	1188-4169/1481-8531	Communicable diseases – Control – Epidemiology	-	Yes	52	1992	Canada	French/English	Text in English and French	
***Category C: Journals dedicated to specific fields of epidemiology***											
*Relevé épidémiologique hebdomadaire*	Weekly Epidemiological Record	0049-8114	World Health Organization-Communicable diseases Epidemiology Vaccination Infectious diseases	-	Yes	52	1928	Switzerland (WHO in Geneva)	French/English	Text in English and French	

^1 ^Only major topics *relevant to epidemiology *are listed here.

^2 ^This refers to the original first issue of the journal despite any changes made to the journal's title or concept in the past.

* The publisher of the *Bulletin of National Academy of Medicine *(of France) is a Dutch publisher.

† Biannual publication.

As shown in Table 1, there are only three Canadian journals covering the field of epidemiology and public health and they are bilingual. As in other non-English-speaking countries, francophone journals including those in France, Switzerland and Africa are struggling to survive considering the fact that English is currently the primary language in the scientific world (Personal communication, Dr. Benoit Gaumer, Associate Professor, Department of Health Administration, University of Montréal). For researchers in Québec and in francophone countries, it is more rewarding to publish their results in English and this tendency is difficult to reverse. One of the consequences of the current situation is the transformation of French journals into bilingual publications. This is the case for the journal *Ruptures*, previously published by the Department of Public Health of the University of Montréal, which ceased publication in May 2007 due to a lack of human and financial resources [19]. Now, *Ruptures *has become *Healthcare Policy/Politiques de Santé *under a more prestigious publishing house, Longwoods (Personal communication, Ms. Saul-Cohen, Department of Health Administration, University of Montréal).

In Africa, there are several French journals available as well as one publication that publishes articles in both English and French (Table 1). Senegal, Madagascar, Mali, Tunisia, Côte d'Ivoire and Nigeria are currently the only African countries that publish epidemiology or public health journals in French. However, one can retrieve (via PubMed) a few other journals from these countries that only offer articles in English or in their native languages.

### Bibliographic databases in French

One of the most extensive bibliographic databases in French is CAT.INIST, which provides access to 15 million references [20]. This database contains documents covering research in Sciences, Technology, Medicine, Humanity and Social Sciences across the world from 1973 to today. CAT.INIST combines documents from The Institute for Scientific and Technical Information (INIST) [21] (English website: [22]) and the Centre national de la recherche scientifique (CNRS) [23] (English website: [24]). Other relevant databases include Base de données Santé Publique (Public Health Database), France [25], Institut national de santé publique (National Public Health Institute), Québec (Canada) [26] and Masson, Éditeur médical et paramédical (Masson, Medical and Paramedical Editor), France [27].

### Professional education in epidemiology and public health in French

Today, France offers post-graduate training through the École nationale de la Santé Publique [28], the Institut de Veille Sanitaire [29] and the Institut Pasteur [13]. In Québec, epidemiology and public health are taught in graduate and post-graduate programmes at the University of Montréal (in French) [30], at Laval University (in French) [31] and McGill University (in English) [32]. Basic epidemiology and public health training is also offered in other universities such as the University of Sherbrooke [33] and University of Québec at Chicoutimi [34]. The Québec National Public Health Institute provides training sessions for practitioners and public health researchers [35]. Through universities and specialist institutes, postgraduate epidemiology and public health courses are also offered in French in Belgium (e.g. Prince Leopold Institute of Tropical Medicine, Antwerp [36]) and Switzerland (cf. Swiss School of Public Health plus [37], a foundation jointly founded by the Universities of Basel, Bern, Geneva, Lausanne, Lugano and Zurich) as well as in francophone Africa (cf. [38] and [39] in which north African countries were classified in a separate category): Algeria (e.g. École National de Santé Publique [40]), Benin (Institut Regional de Santé Publique de Ouidah), Cameroon (University of Yaounde 1), Côte d'Ivoire (UFR des Sciences Medicales de l'Université de Cocody-Abidjan), Democratic Republic of Congo (e.g. Kinshasha School of Public Health), Morocco (Institut National de l'Administration Sanitaire [41]), Senegal (Institut de Santé et Développement [42]) and Tunisia (e.g. Institut National de la Santé Publique). However, there are still 18 francophone African countries without any advanced public health education programmes [38].

### Implications for French-language publications

In conclusion, there is no doubt that the epidemiology and public health journals of francophone countries and Québec are struggling against the dominance of those in English-speaking countries such as the United Kingdom and the United States of America. The fact that English-speaking countries have been dominant in the history, literature and academic field of epidemiology for many years certainly influenced the current scene in francophone countries. French-speaking researchers and practitioners do not have a choice but to refer to English epidemiology and public health journals for their work as they offer more complete and accurate information at the international level.

This fact is ironic considering that France, Québec and francophone Africa are actively providing training in epidemiology and public health in French. Employment opportunities for French researchers and practitioners are flourishing within health care, administration, and academia due to the increase of chronic and infectious diseases. It is possible that partnerships between French-speaking countries and Québec, such as the Observatoire franco-québécois de la santé et de la solidarité (OFQSS) [43] created in 2002 in order to enable Québec and France to distribute their work in the social and sanitary field, will promote better development and visibility of research published in French publications.

## Scenario 2: German

### Background: Germany

Today, German epidemiologists tend to publish major results and methodological aspects of their studies in English language journals (European or American), followed by papers on selective aspects of the study or overviews in German in German journals. For example, a team who conducted a retrospective cohort study of ethnic German migrants in Germany published innovative aspects of the methodology and major findings in English in the *European Journal of Epidemiology *[44], *BMC Public Health *[45] and *European Journal of Cancer *[46], respectively. Thereafter, a summary of the major epidemiological findings appeared in German in the German medical journal *Deutsches Ärzteblatt *(including an English translation, see footnote 3 in Table 2) [47].

**Table 2:** Selection of German, Austrian, and Swiss journals relevant to epidemiology

**Title**	**English translation (for German title)**	**ISSN (printed/electronic)**	**Epidemiological topics covered^1^**	**Official Impact Factor 2006**	**Listed in Medline**	**Issues per year**	**First edition^2^**	**Country of publication**	**Official journal language**	**Language predominantly used**	**URL**
***Category A: Journals covering (mainly non-clinical) epidemiology***											

*Bundesgesundheitsblatt – Gesundheitsforschung – Gesundheitsschutz*	Federal Health Journal – Health Research – Health Protection	1436-9990/1437-1588	Public health epidemiology	-	Yes	12	1958/59	Germany	German		
*Das Gesundheitswesen*	The Health System	0941-3790	Wide range of topics	0.716	Yes	12	1949	Germany	German/English	German	
*International Journal of Public Health (formerly: Sozial- und Präventivmedizin)*		1661-8556/1420-911X	Public health epidemiology	1.013	Yes	6	1900	Switzerland	German/French/English	English	
*Journal of Public Health (formerly: Zeitschrift für Gesundheitswissenschaften)*		0943-1853/1613-2238	Public health epidemiology	-	No	6	1990	Germany	English		
*Public Health Forum. Forschung – Lehre – Praxis*	Public Health Forum. Research – Education – Practice	0944-5587	Public health epidemiology (only brief summaries)	-	No	4	1993	Germany	German		

***Category B: Medical journals occasionally covering (mainly clinical) epidemiology***											

*Deutsche Medizinische Wochenschrift*	German Medical Weekly	0012-0472	Wide range of topics	0.584	Yes	52	1874/75	Germany	German		
*Deutsches Ärzteblatt*	German Physicians' Journal	0012-1207	Wide range of topics	-	Yes	52	1903	Germany	German^3^		
*MMW – Fortschritte der Medizin*	MMW – Progress in Medicine	1438-3276	Clinical (and public health) epidemiology	-	Yes	52	1853/82	Germany	German		
*Swiss Medical Weekly*		1424-7860/1424-3997	Clinical epidemiology	1.346	Yes	52	1871	Switzerland	English	English	
*Wiener Klinische Wochenschrift – The Middle European Journal of Medicine*	Vienna Clinical Weekly	0043-5325/1613-7671	Clinical epidemiology	0.804	Yes	24	1888	Austria	German/English	German and English approx. equal	
*Wiener Medizinische Wochenschrift*	Vienna Medical Weekly	0043-5341/1563-258X	Clinical (and public health) epidemiology	-	Yes	24	1851	Austria	German/English	German and English approx. equal	

***Category C: Journals dedicated to specific fields of epidemiology*^4^**											

*Arbeitsmedizin, Sozialmedizin, Umweltmedizin*	Occupational Medicine, Social Medicine, Environmental Medicine	0944-6052	Occupational health and public health epidemiology	-	No^5^	12	1962	Germany	German	German	
*Dermatologie in Beruf und Umwelt*	Occupational and Environmental Dermatology	1438-776X	Occupational health related to dermatology	-	No	4	1953	Germany	German/English	German	
*Epidemiologisches Bulletin*	Epidemiological Bulletin	1430-0265/1430-1172	Infectious diseases	-	No	52	1995	Germany	German		
*Ernährung & Medizin*	Nutrition & Medicine	1439-1635	Nutrition	-	No	4	1985	Germany	German		

***Category D: Journals dedicated to methodical aspects of epidemiology***											

*GMS Medizinische Informatik, Biometrie und Epidemiologie/GMS Medical Informatics, Biometry and Epidemiology (MIBE) *(formerly: *Informatik, Biometrie und Epidemiologie in Medizin und Biologie*)		1860-9171 (electronic only)	Methods, historical aspects and applications	-	No	3	1969	Germany	German/English	German	

^1 ^Only major topics *relevant to epidemiology *are listed here.

^2 ^This refers to the original first issue of the journal despite any changes made to the journal's title or concept in the past.

^3 ^English translations of articles relevant to medical research appearing from volume 103, no. 37, 2006 onwards are available on the journal's website

^4 ^Since there are many specialized German journals that sporadically publish articles relevant to epidemiology, this section only gives a few examples. Most of these journals publish in German.

^5 ^Indexed by Medline until 1993. Until then published as *Arbeitsmedizin, Sozialmedizin, Präventivmedizin*.

In other words, when German epidemiologists wish to retrieve findings of studies conducted in Germany, they have to access the international epidemiological and public health literature. At the same time, German public health practitioners, who often do not routinely access English-language journals, receive information on German studies belatedly and only in the form of summary papers. What is the history behind this situation?

German used to be a major language of science in the 19th and early 20th century. This also applied to social medicine (the precursor of epidemiology and public health) [48]. For example, Robert Koch published almost exclusively in German [49]. Thus, German scientists used to communicate in German in the past. This tradition still exists in Germany even today, especially in the social sciences but also in medicine [50]. However, after World War I and II, the German language lost some of its dominance in many core areas of science. The reason for this was, among other things, anti-German sentiment related to the atrocities committed during the Nazi regime, as well as the economic and cultural influence of the United States combined with the prestige associated with the English language [48].

After World War II, social medicine was discredited in Germany because of its misuse by the Nazis (e.g. in the field of eugenics) [51]. A renaissance started only in the 1980s and especially the 1990s [52]. German-language medical and medical research journals started to recover earlier, but initially followed a "German", rather than international approach: a tendency to focus on experience, rather than evidence, persisted much longer in the German literature than in that of the United Kingdom and the United States. For example, evidence from randomised, controlled trials was introduced in a systematic way only much later in German-language journals [53].

As will be seen in the following, the historical development of epidemiology also finds expression in the publication behaviour of epidemiologists and the current role of German-language epidemiology and public health publications.

### Journals in epidemiology and public health: German

In Germany, epidemiology is considered to be one of the constitutional or core sciences of public health [52]. Hence, the findings of non-clinical epidemiological studies are published in public health journals as well as in epidemiological journals. Naturally, the former cover a broad range of topics, of which epidemiology is only one.

In Table 2, journals publishing epidemiological papers with a public health focus are grouped in category A. The majority of these journals are published in German, with abstracts in both German and English. Some of these journals are increasingly accepting and publishing English language articles. One example is the *International Journal of Public Health*, formerly *Sozial- und Präventivmedizin*. This journal is in the process of completely switching over to English language, an aim which the *Journal of Public Health *(formerly *Zeitschrift für Gesundheitswissenschaften*) has already achieved.

Clinical epidemiological research is occasionally published in general medical journals. Examples are listed under category B in Table 2. These journals are almost exclusively in German. Findings from clinical epidemiology can also be found in journals dedicated to subfields of medicine such as occupational health (see category C in Table 2 for some such examples). Some of these journals accept papers in English, e.g. the journal with the German title *Dermatologie in Beruf und Umwelt *(*Dermatology in the Workplace and Environment*).

Besides the actual subject matter, epidemiological studies often comprise innovative methodological aspects, which are usually covered by more specialized epidemiological journals. Only one such journal, *German Medical Science (GMS) Medizinische Informatik, Biometrie und Epidemiologie *(see category D in Table 2) is published in the German language. Incidentally, this journal is the only journal in the table featuring "epidemiology" in its (sub-)title. However, this journal is published only three times a year and contains only a few articles per issue.

In addition, there is a tendency in Germany today to assume that excellence in science is reflected by publications in English, rather than in German. This could be due to the higher impact factors of English language journals and the higher "international visibility" of English-language publications. This phenomenon can be evidenced in the so-called "Exzellenzinitiative" in which German universities competed for government funding – "international visibility" was a crucial criterion in the evaluation of applications [54]. Clearly, English-language publications ensure a higher international visibility than German-language ones.

In public health, however, many scientists still write in German. It is striking to observe to what a low extent English language public health papers – even those in high impact journals – are read and discussed in Germany, compared to German language publications in journals that are not even listed in Medline. This has a peculiar side effect: public health specialists and epidemiologists in Germany who publish mainly in English are sometimes better known in neighbouring European countries than they are in Germany itself. They only become more widely known and acknowledged in their home country and language after publishing overview articles in widely read German journals (as in the example above).

### Bibliographic databases in German

As is the case for Italian and French, there are also various web-based bibliographic databases in German speaking countries that also cover literature relevant to public health and epidemiology. Aside from general catalogues offered by national and university libraries in Austria, Switzerland, and Germany, some examples that focus on medicine, social and health sciences in particular include (topics covered are given in brackets): *SOMED *(social medicine) [55], *Archido *(publications and information related to drugs and addiction) [56], *BELIT *(Bioethics) [57], *ETHMED *(Ethics in medicine) [58], *GeroLit *(Gerontology) [59], resources offered by the *Federal Centre for Health Education (Bundeszentrale für gesundheitliche Aufklärung, BZgA) *(portal for different databases relevant to prevention and health promotion) [60], *Zeitschriften-Dokumentation Sozialwesen/Pflege *(social services, nursing) [61]. Additionally, some services offer meta-search in different databases. Two examples are the search engines offered by the German Institute of Medical Documentation and Information (DIMDI) [62] and MedPilot [63], a service offered by a cooperation of different institutions. Most of the databases mentioned above are not limited to German language publications but also cover international literature. Apart from these bibliographic collections, web-based surveillance information systems and other web-based resources relevant to health reporting are available in all three countries.

### Professional education in epidemiology and public health in Germany, Austria, and Switzerland

Until the late 1990s, there were no postgraduate epidemiology training courses in Germany. The first MSE (Master of Science in Epidemiology) course only started in 2001. In the early- to mid-1990s there was a special programme ("Sonderprogramm Epidemiologie") funded by the government to send young German scientists to the United Kingdom or the United States for training in epidemiology. Many of those who graduated from international courses later took up leadership positions in German academia (e.g. chairs in epidemiology) and routinely write their major epidemiological papers in English. Thus, the "Sonderprogramm" contributed to the trend among German epidemiologists to publish mainly in English.

Due to a similar influence of National Socialism, the historic development of public health in Austria bears a similar pattern to that of Germany. Hence, public health in Austria also has a rather short tradition. Before the establishment of public health courses in recent years, public health education took place abroad and was supported by scholarship programs. The oldest German-language School of Public Health is located in Austria and was founded in 1986 [64].

In Switzerland, however, the development of public health started a bit earlier. In the 1950s and 1960s, some Swiss physicians took epidemiology and public health courses in the United States and the United Kingdom, mostly at their own expense. Later, further education in public health was financed through scholarships via a national fund. Public health courses have been available in Switzerland since 1990 [65].

## Scenario 3: Italian

### Background: Italy

In Italy, before the unification in 1861, epidemiology developed within the context of public health [66]. In particular, based on either the miasmatic or the epidemiological-materialistic paradigm of epidemics, scientists developed theories to interpret the cholera outbreaks periodically striking the Italian peninsula [67-69]. These efforts contributed to the identification of the *Vibrio cholerae *bacillus in Florence by Filippo Pacini in 1854 [70].

Following the unification, epidemiology strongly contributed to the major public health efforts devoted to control malaria which affected most of the country. Within this context descriptive and analytical epidemiological methods were adopted to assess the local burden of malaria, to design and test the efficacy and impact of both preventive and curative measures, and finally to evaluate the results of extensive campaigns against malaria in rural settings. Eventually, Italy was declared malaria-free in 1962 [71].

After the Second World War, the focus progressively shifted towards non-communicable diseases; in particular, major efforts were devoted to occupational, environmental and cancer epidemiology. The contribution of Italian epidemiology at an international level has become increasingly relevant. As an example we would like to recall the key role played by Lorenzo Tomatis (1929–2007), director of the International Agency for Research on Cancer (IARC) between 1982 and 1993, in setting up the IARC Monographs on the Evaluation of Carcinogenic Risks to Humans programme, which seeks to identify the causes of human cancer [72].

During the last few decades, epidemiology has become increasingly relevant in Italy as a driving force of the National Health Service. Epidemiological resources and activities are currently present both at the central level, in particular within the National Health Institute (Istituto Superiore di Sanità or ISS) [73], and at a peripheral level, in particular within agencies and observatories present in the twenty Italian regions. These central and peripheral epidemiological and public health activities are coordinated by the National Center for Disease Control (CCM) [74]. Furthermore, since the Italian National Health Service is largely run at a regional level, a substantial amount of epidemiological activity is also carried out at smaller – e.g. Local Health Unit – levels. A typical example is given by the 21 population-based cancer registries active mainly in Northern and Central Italy [75], covering about the 25% of total Italian population [76].

### Journals in epidemiology and public health: Italian

Similar to what happens in other non-English speaking countries, Italian epidemiologists aim to publish major results [77] and methodological aspects of their investigations in international journals [78], whereas selected reviews and studies with a predominant local interest are published in Italian journals. As shown in Table 3, a number of Italian epidemiological and public health journals are indexed in Medline with abstracts available in English. In particular, the journal *Epidemiologia & Prevenzione *regularly publishes monothematic supplements on selected topics such as health consequences of air pollution, cancer statistics and screening program management. Recently, the tendency to publish (or translate) selected articles in English has emerged, while two new public health journals have adopted English as their official language.

**Table 3:** Selection of Italian journals relevant to epidemiology

**Title**	**English translation (for Italian title)**	**ISSN (printed/electronic)**	**Topics**	**Official Impact factor 2006**	**Listed in Medline**	**Issues per year**	**First edition**	**Language**	**URL**
***Journals covering epidemiology and public health***									

Annali dell'Istituto Superiore di Sanità	Annals of Istituto Superiore di Sanità	0021-2571	Public health	-	Yes	4	1965	English	
Annali di Igiene	Annals of Hygiene	1120-9135	Public health Microbiology	-	Yes	6	1889*	Italian	
Epidemiologia & Prevenzione	Epidemiology and prevention	1120-9763	Epidemiology	#	Yes	6	1976	Italian	
Epidemiologia e Psichiatria Sociale	Epidemiology and Social Psychiatry	1121-189X	Epidemiology Psychiatry	#	Yes	4	1992	Italian	
Igiene e Sanità Pubblica	Hygiene and Public Health	0019-1639	Public health	-	Yes	6	1945	Italian	
Igiene Moderna	Modern Hygiene	0019-1655	Public health	-	No	12	1908	Italian	
Italian Journal of Public Health		1723-7807/1723-7815	Public health	-	No	4	2003	English	
Journal of Preventive Medicine and Hygiene		1121-2233	Public health	-	Yes	4	1989	English	

***A selection of specialty journals frequently covering epidemiological issues***									

La Medicina del Lavoro	Occupational Medicine	0025-7818	Occupational health	#	Yes	6	1901	Italian	
Giornale Italiano delle Infezioni Ospedaliere	Italian Journal of Nosocomial Infections	1122-407X	Nosocomial infections	-	No	4	1994	Italian	
Giornale Italiano di Farmacia Clinica	Italian Journal of Clinical Pharmacology	1120-3749	Clinical pharmacology	-	No	4	1987	Italian	
Giornale Italiano di Medicina del Lavoro ed Ergonomia	Italian Journal of Occupational Medicine and Ergonomics	1592-7830	Occupational health	-	Yes	4	1979	Italian	
Tumori	Neoplasms	0300-8916	Oncology	0.701	Yes	6	1911	Italian/English	

* *Annali di Igiene *was first founded as *Annali dell'Istituto d'Igiene Sperimentale della R. Universita di Roma*, and had undergone a number of title changes. It was under its current title since 1989.

# Included in the Web of Science; Impact Factor due in the forthcoming years.

While there is no widely circulated Italian medical journal addressing topics relevant to general medicine, many specialty journals often publish epidemiological papers relevant to their domain of interest. The topics addressed by these journals encompass most of the medical specialties such as mental health, occupational health, nosocomial infections, oncology and clinical pharmacology.

One major weakness of Italian epidemiological and public health journals arises from their limited local readership. In particular, these journals are read by a small proportion of public health practitioners, while they are largely ignored by physicians and other medical practitioners. The lack of a common methodological approach, conflicting research interests, and the inability to ensure dissemination of epidemiological data at the local level results in poor communication between public health services, the academic sector, and experts involved in clinical epidemiology. The inability to reach a suitable audience challenges the reason for keeping Italian as the main publishing language: if a relevant proportion of public health practitioners do not access local publications, these become non-relevant to the health care development process.

### Databases and online resources: Italian

Although no comprehensive bibliographic database is available in Italian, many databases relevant to epidemiology and public health are accessible on the web. Without claiming any completeness, the following websites can be cited: a registry of clinical (drug) trials conducted in Italy [79]; an overview of epidemiological activities (studies, meeting, resources) throughout the country [80]; data on health care delivery (including hospital discharges by primary diagnosis and intervention, and infectious diseases with mandatory notification) [81]; general country statistics (demography, socio-economic indicators, mortality); an Italian version of the Health for All Database [82]; and incidence, mortality and survival from the Italian cancer registries [75]. Finally, information from the Italian Environmental Protection Agency is accessible at its own website [83].

### Professional education in epidemiology in Italy

At the academic level, epidemiology is regularly taught in under- and post-graduate courses as part of the core scientific syllabus. Moreover, basic epidemiological training is often required to write and defend the thesis concluding health-related academic courses. It is worth noting that the intensive summer school "European Educational Programme in Epidemiology" (EEPE) [84] has been held in Florence since 1988, and has been attended by several hundred students from European and extra-European countries [7]. The first post-graduate course in Epidemiology, a Masters of Science (M.Sc.) in Epidemiology, was started in 1997 by the Italian Association of Epidemiology (AIE) [85], is now run by the University of Turin on a biennial basis, and is part of the network "European Master of Science in Epidemiology" (EU-MSE) [86]. Currently, four additional M.Sc. programmes and several short courses are available through the country [80,85].

## Discussion in a comparative perspective

This paper gave an overview and analysis of epidemiological and public health journals, databases and professional education represented in three Western European languages: French, German and Italian. The historical development of the profession of epidemiology and public health and their respective journals, databases and professional education in these linguistic communities followed different paths due to differences in historical, social and political circumstances.

Bibliographic databases are gateways to the vast quantity of medical literature. There is evidence that many non-English medical journals are not indexed by major English-based bibliographic databases like PubMed and Web of Science, and that alternative databases like EMBASE provide more comprehensive coverage of non-English literature (please refer to the following papers, all available in this thematic series: papers on Chinese bibliographic databases [87,88], on LILACS and SciELO databases for Spanish and Portuguese [10,11], and on how EMBASE enhances access to randomised control trials [89]). While bibliographic databases that cover medical and scientific journals are available in French and German, their absence in Italian may reflect the relative dearth of Italian academic journals, but is possibly more likely a function of a lack of funding or commitment from the Italian government. However, various online databases and web resources are available in all three languages to provide interfaces through which epidemiological and public health information can be further disseminated in a national or regional context.

Academic literature in a given language presupposes a professional community who will read and contribute to it. The strength of a language as a medium of communication in epidemiology and public health is reflected, to a certain extent, in professional education in the respective language. France and other French-speaking countries or territories have a long tradition of public health professional education in French. Thus, they have fostered a community of French-speaking epidemiologists and public health practitioners who in turn can contribute to the professional literature in French. The relatively late development of professional education in modern epidemiology in the German-speaking countries and Italy may have contributed further to the contemporary dilemma of epidemiology and public health journals published in these languages.

Against the backdrop of English being the contemporary *lingua franca *of the global scientific community today, both practitioners and researchers in these fields face the difficult dilemma of switching to English and forsaking their native tongues as their means of scientific communication, or holding onto their national scientific heritage and in the process possibly losing out in the race of competitive international scientific research. However, it would be biased to ignore the fact that a globalising world, not least the scientific community at large, demands a global language so as to break down linguistic barriers and foster international communication in a more effective and efficient way. As a legacy of the British Empire and the current status of the USA as the leading superpower, English has overtaken French and German as the global language [90]. In a nutshell, it is a matter of prioritising efficiency versus equity [91].

In a market economy, it is the size of readership that determines the circulation and thus the survival of a journal. The larger the size and the more international the potential readerships are, the more likely a journal is to survive. As a legacy of the French and Belgian colonial empires, French is an official language in 30 countries [92,93], and may survive the globalisation of English as a means of scientific communication. However, given the continual growth of English as a language of international communication and the fact that publication in English gains a wider circulation and a better chance of being cited, the trend of even French-speaking scientists now switching to publish in English in order to compete in the international scene is obvious and difficult to reverse [94]. (The Appendix provides a brief analysis of English-language epidemiological and public health journals indexed in PubMed.)

Sources of funding may also be a related issue. Given that many international epidemiological and public health research programmes are now funded by international agencies and foundations as well as governments of some Anglophone countries, it is likely that research outputs from these programmes will be required to be published in English in order to appeal to as wide and international an audience as possible.

In addition, it is important to take notice of the trend of a growing division between researchers and practitioners and the corresponding classification of journals into international and local focus. It seems that in all three linguistic communities mentioned in this paper, researchers tend to read and publish in English, unlike practitioners who mainly read in their native languages. This dichotomy of international English language research journals and local native language journals for continuing medical education has made an impact on the practice of the profession itself, e.g. by making it necessary to facilitate the publication of reviews in the native language 'local' journals of the latest studies published in English in international journals. Indeed, the publication of reviews in the native language should have the function of encouraging physicians, other health-care or social services professionals, policy makers and stakeholders (e.g. patient associations, coalitions against environmental risks and trade unions, etc.) to access the latest research outputs. Incidentally, this phenomenon of two tiers of journals is observed in Russia as well (cf. the paper by Vlassov and Danishevskii [95] in this thematic series).

Nevertheless, as Ofori-Adjei et al. argue, local journals in native languages are a health resource and are important for the contextualisation of evidence on which public health practices are based [50]. Thus as epidemiologists, it is important to ensure that data published in non-English journals (especially when we conduct meta-analyses and systematic reviews) are not overlooked. Furthermore, the importance of channelling the knowledge of research outputs published in English into our respective linguistic communities through writing reviews and summaries in the local native language journals should be appreciated and encouraged. Considering the information presented in this article, it can be ultimately stated that language does matter a great deal in epidemiology today.

## Abstract in alternative languages

The abstract of this editorial has been translated into the following languages by the following translators (names in brackets):

• Chinese – simplified characters (Mr. Isaac Chun-Hai Fung and Dr. Yan Che) [see Additional file 1]

• Chinese – traditional characters (Mr. Isaac Chun-Hai Fung and Dr. Yan Che) [see Additional file 2]

• French (Mr. Philip Harding-Esch) [see Additional file 3]

• German (Mr. Patrick Brzoska and Prof. Oliver Razum) [see Additional file 4]

• Italian (Dr. Iacopo Baussano and Dr. Ugo Fedeli) [see Additional file 5]

• Spanish (Ms. Gabriela Gomez) [see Additional file 6]

## Appendix: Epidemiological and public health journals in English

Data on epidemiological and public health journals published in English were retrieved from PubMed Journals Database on 4 December 2007 using this combination of keywords: (Public health OR epidemiol* OR hygiene). A total of 447 journal records were retrieved, of which 247 titles are still being published (No entry of Publication End Year). Among these 247 titles, 173 are in English (70%) while 74 (30%) are in other languages or multiple languages (including English). Among the 173 English journals, 143 (82.66%) are published in English-speaking countries (Australia, Canada, England, Ireland, New Zealand, Scotland, South Africa and the United States). Among the 30 English journals published in non-English-speaking countries, nine are published in the Netherlands, four in Switzerland, three in Germany, three in Italy, and one in China, Czech Republic, Denmark, Egypt, India, Japan, Sweden, Tanzania, Thailand and Uganda respectively.

## Competing interests

The authors declare that they have no competing interests.

## Authors' contributions

ICHF conceived the original ideas of the project and organised the other co-authors to contribute to this paper. He also wrote the first draft of the introductory and discussion sections and the Appendix. CL wrote the French section and Table 1. OR and PB wrote the German section and Table 2. IB and UF wrote the Italian section and Table 3. All authors revised the final manuscript and agree to the publication of this paper in its final form.

## Supplementary Material

Additional File 1 Abstract in Chinese – simplified characters. Click here for file

Additional File 2 Abstract in Chinese – traditional characters. Click here for file

Additional File 3 Abstract in French. Click here for file

Additional File 4 Abstract in German. Click here for file

Additional File 5 Abstract in Italian. Click here for file

Additional File 6 Abstract in Spanish. Click here for file
