# Supplementary material for: Does language matter? A case study of epidemiological and public health journals, databases and professional education in French, German and Italian
Source: Emerg Themes Epidemiol. 2008 Sep 30;5:16. doi: 10.1186/1742-7622-5-16 (PMC2570667; doi:10.1186/1742-7622-5-16)
Supplement: Additional File 1 — Abstract in Chinese – simplified characters. [file 1742-7622-5-16-S1.pdf]

Simplified Chinese / 简体中文

分析透视

语言是个问题吗？法语、德语和意大利语的流行病学及公共卫生期刊、数据库及专业教育的个案研究

作者：Iacopo Baussano, Patrick Brzoska, Ugo Fedeli, Claudia Larouche, Oliver Razum, 冯俊熙(Isaac Chun-Hai Fung)

摘要

流行病学和公共卫生通常因所处环境不同而各异。不同国家以不同语言出版的期刊一方面可以作为数据的来源，另一方面也是将证据与当地公共卫生实践相整合的重要渠道。这些数据库以不同语言作为载体，让访问相关期刊变得更加容易，而以这些语言进行专业教育，则又促进了当地流行病学及公共卫生专业技术的发展。然而，在全球化的世代里，英语已成为科学交流的通用语言，许多以非英语语言出版的期刊正面临两难的处境：转用英语出版，将面对全球竞争；沿用本地语言，读者群却局限于本地。本文分析了三种西欧语言——法语、德语和意大利语——流行病学的历史发展，以及当前该三种语言流行病学及公共卫生期刊、数据库和专业教育的处境，并探讨了它们今天存在的动力与困境。

（中文摘要翻译：冯俊熙、车　焱）
