# Supplementary material for: Does language matter? A case study of epidemiological and public health journals, databases and professional education in French, German and Italian
Source: Emerg Themes Epidemiol. 2008 Sep 30;5:16. doi: 10.1186/1742-7622-5-16 (PMC2570667; doi:10.1186/1742-7622-5-16)
Supplement: Additional File 2 — Abstract in Chinese – traditional characters. [file 1742-7622-5-16-S2.pdf]

Traditional Chinese / 繁體中文

分析透視

語言是個問題嗎？法語、德語和意大利語的流行病學及公共衛生期刊、數據庫及專業教育的個案研究

作者：Iacopo Baussano, Patrick Brzoska, Ugo Fedeli, Claudia Larouche, Oliver Razum, 馮儁熙(Isaac Chun-Hai Fung)

摘要

流行病學和公共衛生通常因所處環境不同而各異。不同國家以不同語言出版的期刊一方面可以作為數據的來源，另一方面也是將證據與當地公共衛生實踐相整合的重要渠道。這些數據庫以不同語言作為載體，讓訪問相關期刊變得更加容易，而以這些語言進行專業教育，則又促進了當地流行病學及公共衛生專業技術的發展。然而，在全球化的世代裡，英語已成爲科學交流的通用語言，許多以非英語語言出版的期刊正面臨兩難的處境：轉用英語出版，將面對全球競爭；沿用本地語言，讀者群卻局限於本地。本文分析了三種西歐語言——法語、德語和意大利語——流行病學的歷史發展，以及當前該三種語言流行病學及公共衛生期刊、數據庫和專業教育的處境，並探討了它們今天存在的動力與困境。

（中文摘要翻譯：馮儁熙、車　焱）
