# Supplementary material for: Does language matter? A case study of epidemiological and public health journals, databases and professional education in French, German and Italian
Source: Emerg Themes Epidemiol. 2008 Sep 30;5:16. doi: 10.1186/1742-7622-5-16 (PMC2570667; doi:10.1186/1742-7622-5-16)
Supplement: Additional File 3 — Abstract in French. [file 1742-7622-5-16-S3.pdf]

French / Français

Perspectives analytiques

Publier oui, mais en quelle langue ? Une étude de cas de revues, de bases de données et de formation professionnelle en épidémiologie et en santé publique en français, en allemand et en italien.

Auteurs : Iacopo Baussano, Patrick Brzoska, Ugo Fedeli, Claudia Larouche, Oliver Razum, Isaac Chun-Hai Fung

Résumé

L'épidémiologie et la santé publique sont normalement spécifiques à leur contexte. Les revues publiées dans des langues et dans des pays différents jouent un double rôle comme sources de données d'une part, et d'autre part comme voies par lesquelles les évidences sont incorporées aux pratiques locales en santé publique. Les bases de données publiées dans ces langues facilitent l'accès aux revues pertinentes, et la formation professionnelle dans ces langues facilite le développement dans les pays en question de l'expertise en épidémiologie et en santé publique. Cependant, au fur et à mesure que l'anglais est devenu la lingua franca de la communication scientifique à l'ère de la globalisation, grand nombre des revues publiées dans des langues autres que l'anglais font face au dilemme de passer à l'anglais pour faire concurrence au niveau international, ou de garder la langue nationale et d'avoir une circulation limitée à des lecteurs locaux. Cet article analyse le développement historique

de l’ épidémiologie ainsi que l’état actuel des revues, des bases de données et de la formation professionnelle en épidémiologie et en santé publique en trois langues d’Europe occidentale : le français, l’allemand et l’italien. Il examine également les dynamiques et les difficultés existant aujourd’hui dans ces domaines.

Traduit de l’anglais par Philip Harding-Esch
