# Supplementary material for: Does language matter? A case study of epidemiological and public health journals, databases and professional education in French, German and Italian
Source: Emerg Themes Epidemiol. 2008 Sep 30;5:16. doi: 10.1186/1742-7622-5-16 (PMC2570667; doi:10.1186/1742-7622-5-16)
Supplement: Additional File 4 — Abstract in German. [file 1742-7622-5-16-S4.pdf]

German / Deutsch

Analytische Perspektive

Welche Rolle spielt Sprache? Eine Fallstudie an französisch-, deutsch- und italienischsprachigen Fachzeitschriften, Datenbanken und Ausbildungsmöglichkeiten in Epidemiologie und Public Health.

Autoren: Iacopo Baussano, Patrick Brzoska, Ugo Fedeli, Claudia Larouche, Oliver Razum, Isaac Chun-Hai Fung

Zusammenfassung

Epidemiologie und Public Health werden jeweils in einem spezifischen lokalen Kontext betrieben. Daher spielen Fachzeitschriften, die in der jeweiligen Lokalsprache publiziert werden, eine wichtige Rolle als Informationsquellen sowie als Medien, durch die neue Erkenntnisse Einzug in die Praxis von Public Health halten. Datenbanken in den jeweiligen Sprachen erleichtern den Zugang zu relevanten Zeitschriften und eine muttersprachliche Ausbildung fördert die einheimische Expertise in Epidemiologie und Public Health. Mit der zunehmenden Bedeutung des Englischen als Wissenschaftssprache in Zeiten der Globalisierung stehen viele nicht englischsprachige Zeitschriften aber vor dem Dilemma, ihre Publikationssprache entweder auf Englisch umzustellen, um im internationalen Wettbewerb bestehen zu können, oder aber die jeweilige Landessprache beizubehalten und sich auf einen kleineren Leserkreis zu beschränken. Dieser Artikel beschreibt die geschichtliche Entwicklung von Epidemiologie und beschäftigt sich mit französisch-, deutsch- und italienischsprachigen Zeitschriften,

Datenbanken und Ausbildungsmöglichkeiten in Epidemiologie und Public Health und untersucht die Dynamik und Schwierigkeiten, denen diese drei westeuropäischen Sprachen in diesem Fachgebiet heute ausgesetzt sind.

Übersetzung durch Patrick Brzoska und Oliver Razum
