# Supplementary material for: Does language matter? A case study of epidemiological and public health journals, databases and professional education in French, German and Italian
Source: Emerg Themes Epidemiol. 2008 Sep 30;5:16. doi: 10.1186/1742-7622-5-16 (PMC2570667; doi:10.1186/1742-7622-5-16)
Supplement: Additional File 5 — Abstract in Italian. [file 1742-7622-5-16-S5.pdf]

Italian / Italiano

Articolo di analisi

Quale importanza ha la lingua? Una presentazione di riviste, database e percorsi formativi in epidemiologia e sanità pubblica in francese, tedesco e italiano

Autori: Iacopo Baussano, Patrick Brzoska, Ugo Fedeli, Claudia Larouche, Oliver Razum, Isaac Chun-Hai Fung

Abstract: L’epidemiologia e la sanità pubblica sono legate al contesto in cui operano, così le riviste pubblicate in differenti paesi e lingue svolgono un ruolo sia di fonte di dati che di canali attraverso cui le evidenze sono incorporate nelle pratiche locali di sanità pubblica. I database in lingua locale agevolano l’accesso alle riviste pertinenti, e le proposte formative facilitano la crescita di competenze locali in epidemiologia e sanità pubblica. D’altra parte, poiché l’inglese è divenuto la *lingua franca* della comunicazione scientifica nell’epoca della globalizzazione, molte riviste pubblicate in lingue differenti dall’inglese si trovano di fronte alla difficile scelta se adottare l’inglese ed assumere un profilo internazionale o rimanere fedeli alla lingua nativa ed avere una circolazione limitata ed un pubblico locale. L’articolo riassume brevemente lo sviluppo storico dell’epidemiologia in tre aree linguistiche dell’Europa occidentale (francese, tedesca ed italiana), ne analizza le dinamiche ed i conflitti e presenta lo scenario attuale in termini di riviste, database e proposte formative in epidemiologia e sanità pubblica.

Tradotto da Iacopo Baussano e Ugo Fedeli
