# Supplementary material for: Does language matter? A case study of epidemiological and public health journals, databases and professional education in French, German and Italian
Source: Emerg Themes Epidemiol. 2008 Sep 30;5:16. doi: 10.1186/1742-7622-5-16 (PMC2570667; doi:10.1186/1742-7622-5-16)
Supplement: Additional File 6 — Abstract in Spanish. [file 1742-7622-5-16-S6.pdf]

Spanish / Español

Perspectiva analítica

¿Importa el idioma? Un estudio de revistas epidemiológicas y de salud pública, bases de datos y formación profesional en francés, alemán e italiano.

Autores: Iacopo Baussano, Patrick Brzoska, Ugo Fedeli, Claudia Larouche, Oliver Razum, Isaac Chun-Hai Fung

Resumen

La Epidemiología y la salud pública generalmente son disciplinas específicas a su contexto. Las revistas publicadas en diferentes idiomas y países tienen un papel importante tanto como fuentes de datos como vías por las cuales la evidencia científica puede ser incorporada dentro de la práctica local de la salud pública. Las bases de datos en estos idiomas facilitan el acceso a las revistas de relevancia local, así como la formación profesional en estos idiomas facilita el desarrollo de una experiencia en epidemiología y salud pública local. Sin embargo, considerando que el inglés se ha vuelto la *lingua franca* de la comunicación científica en la era de la globalización, muchas revistas publicadas en otros idiomas que el inglés se ven confrontadas con el dilema de cambiar a este idioma y competir a nivel internacional o seguir publicando en la lengua nativa y tener una circulación limitada a los lectores locales. Este artículo

analiza el desarrollo histórico de la epidemiología así como la situación de las revistas epidemiológicas y de salud pública, bases de datos y formación profesional en tres idiomas de Europa Occidental: francés, alemán e italiano. Asimismo examina la dinámica y los retos que las confrontan hoy en día.

Traducido por Gabriela Gomez
